# Correlation of Conductive Hearing Impairment With Sizes of Adenoids in the Pediatric Age Group: An Observational Case-Control Study

**DOI:** 10.7759/cureus.44439

**Published:** 2023-08-31

**Authors:** Ashish Khadgi, Krishna Koirala, Sanjay Maharjan, Kamana Chalise, Inclub Dhungana, Bikram Babu Karki

**Affiliations:** 1 Department of Otolaryngology, Head and Neck Surgery, Buddha International Hospital, Ghorahi, NPL; 2 Department of Otolaryngology, Head and Neck Surgery, Manipal College of Medical Sciences, Pokhara, NPL; 3 Department of Otolaryngology, Head and Neck Surgery, Tulsipur City Hospital, Tulsipur, NPL; 4 Department of Otolaryngology, Head and Neck Surgery, BP Koirala Memorial Cancer Hospital, Chitwan, NPL; 5 Department of Otolaryngology, Head and Neck Surgery, Hetauda Hospital, Hetauda, NPL; 6 Department of Otolaryngology, Head and Neck Surgery, Fishtail Hospital, Pokhara, NPL

**Keywords:** pediatric hearing loss, tympanometry, pure tone audiometry, otitis media with effusion, conductive hearing loss

## Abstract

Introduction: When adenoids enlarge and elicit symptoms it is referred to as symptomatic adenoids, which is a preventable cause of hearing loss and nasal obstruction in the pediatric age group. This study was done to correlate conductive hearing loss with the size of adenoids and to emphasize the importance of screening in the pediatric age group.

Methods: An observational case-control study was conducted to analyze the degree of hearing impairment in children with adenoid hypertrophy. In total, 98 patients with at least one symptom of adenoid hypertrophy aged between 5 and <15 years were recruited. Those with conductive hearing loss were in the case group and those with normal hearing were in the control group. Audiometry, tympanogram, X-ray, and fibreoptic nasal endoscopy were conducted and compared.

Results: The mean age of presentation of conductive hearing loss with adenoids was 7.67 years. The mean conductive hearing loss on audiometry was 31.69 dB. Tympanogram showed a type B curve in 40.81% of ears and type C in 26.53%. On X-ray nasopharynx, the majority of cases had grade III hypertrophy followed by grade II and grade IV. In nasal endoscopy, most cases had second-degree adenoid hypertrophy followed by third degree, first degree, and then fourth degree. The highest degree of hearing loss of 32-48 dB was present with fourth-degree adenoids. Conductive hearing loss was five times more in patients with third- and fourth-degree adenoids.

Conclusions: In our study, adenoid hypertrophy has a positive correlation with conductive hearing loss in pediatric patients. So proper screening and early management should be done to prevent hearing loss in children.

## Introduction

The growth of adenoids occurs rapidly in children and stops with little or no change between two and 14 years. In most patients, size diminishes rapidly up to 15 years of age. Upper respiratory tract infection may lead to the multiplication of lymphoid follicles increasing the size of adenoids [1]. Adenoid hypertrophy may lead to obstructive sleep apnoea, ear problems, mouth breathing, failure to thrive, pulmonary hypertension, and craniofacial anomalies which are preventable [2]. Eustachian tube (ET) obstruction in patients with adenoids, may lead to otitis media with effusion (OME) and conductive hearing loss (CHL) of variable degree [3,4].

Hearing loss due to adenoids may go unnoticed which is easily treatable by medical and surgical modalities. To prevent complications from adenoids, early diagnosis and treatment are needed [2]. There is very little evidence of the prevalence of adenoid hypertrophy leading to CHL in children in Nepal so this study is intended to find out the correlation of CHL with the size of adenoids in the pediatric age group in Nepal.

## Materials and methods

This is an observational case-control study done in a tertiary hospital in Nepal between 2nd January 2017 to 2nd January 2018. Ethical approval was received from the Institutional Review Committee and written with verbal consent was taken from the guardians of participating patients. Children presenting with at least one of the symptoms of adenoid hypertrophy such as hearing impairment, mouth breathing, nasal obstruction, snoring, drooling of saliva, sleep disturbance, voice change, headache, or epistaxis were categorized as symptomatic adenoids and were included in the study.

Inclusion and exclusion criteria

Children between 5 and <15 years of age with at least one symptom of adenoids were included in the study after obtaining informed and written consent from their guardians. On the other hand, children between 5 and <15 years of age with no symptoms of adenoids, those with tympanic membrane perforation, a history of frank discharge from the ear, with sensorineural/mixed hearing loss, with a previous history of ear surgery or adenoidectomy, with known congenital diseases, syndromes or concomitant systemic disease and those with any known case of malignancy were excluded from the study.

The sample size was calculated using the following formula and purposive sampling was done:

N = Z2×P×Q/E2

Where P = prevalence of adenoids in children from the data collected from the outpatient department of last year (6.9%; 0.069); Q = 1-P (1-0.069; 0 .931); E = allowable error (5%; 0.0); Z (critical value) = 1.96; N = (1.96)2 x 0.069 x 0.931/0.05 x 0.05 (98.71).

In total, 49 children with at least one symptom of adenoid hypertrophy with CHL in one or both ears shown by audiometry aged 5 - <15 years were included in the case group. Subsequently, children with at least one symptom of adenoid hypertrophy (hearing impairment, mouth breathing, nasal obstruction and discharge, snoring, drooling of saliva, sleep disturbance, voice change, headache, earache, and/or epistaxis) without CHL aged between 5 and <15 years were included in the control group. The case-to-control ratio was taken at 1:1. All patients were sent for tympanometry, X-ray soft tissue nasopharynx lateral view, and fibreoptic nasal endoscopy. Data was collected and analysed using Statistical Package for Social Science (SPSS) v. 25 (IBM Corp., Armonk, NY). The significance level was assessed by calculating the p-value. The p-value was assumed to be less than 0.05.

## Results

A total of 906 children aged between 5 and <15 years attended the outpatient department of a tertiary hospital in Nepal over the study period. The majority of cases belonged to the age group 5-7 years while the age group 11 - < 15 years had the least amount of cases. The mean age of presentation was 7.67 years. It was interesting to note that in the lower age group of 5-7 years, the presence of symptomatic adenoids was more in the case group than in the control group, but in the higher age group of 11-15 years, the presence of symptomatic adenoids was more in the control group than in the case group. There was male predominance in both the case (65%) and control groups (61 %). The male-to-female ratio was 1.8:1 in the case group and 1.5:1 in the control group respectively.

Hearing impairment, nasal obstruction, mouth breathing, and snoring were the most common symptoms. More symptoms were present in the case group than in the control group with the exception of sleep disturbance and earache. The chi-square test was applied and it was found that there was a statistically significant difference in symptoms such as hearing impairment, mouth breathing, nasal obstruction, snoring, headache, and earache between the case and control groups with a p-value less than 0.05. There was no significant difference in symptoms like drooling of saliva, sleep disturbance, voice change, and epistaxis between the case and control groups. 

While studying both ears in the case group, i.e. 98 ears, 69 (70.40%) ears had some degree of CHL while 29 (29.60%) ears had normal hearing (Table 1).

**Table 1:** Hearing status of both ears in cases by audiometry

Status of hearing	No. of ears	Percentage of ears
Normal hearing	29	29.60%
Hearing loss	69	70.40%

Out of the 69 ears with hearing loss, 67 (97.10%) ears had CHL of 26-40 dB and 2 (2.89%) ears had CHL of 41-55 dB (Table 2). The mean CHL on audiometry was 31.69 dB.

**Table 2:** Degree of conductive hearing loss in the case group

Degree	No. of ears	Percentage of ears
26 – 40dB	Right: 30	43.47%
	Left: 37	53.62%
41 – 55dB	Right :1	1.4%
	Left: 1	1.4%
Total	69	100%

Type A tympanogram was present in 32 (32.65%) of case ears and 75 (76.53%) of control ears. Type B tympanogram was present in 40 (40.81%) case-group ears while there was no type B tympanogram in control-group ears. Type C tympanogram was present in 26 (26.53%) of case-group ears and 23 (23.46%) of control-group ears. From the tympanometry findings, we can see that the majority of control-group ears had type A tympanograms while the majority of case-group ears had type B tympanograms indicating fluid in the middle ear. Type C tympanogram was present in the majority of the case-group ears leading to hearing loss in the participants.

In X-ray nasopharynx lateral view, the majority of the case group had grade III hypertrophy in contrast to the control group in which the majority had grade II hypertrophy (Table 3). The most important difference is there was grade IV hypertrophy in eight children in the case group; this was only there in one child in the control group (as per the Clemens et al. classification of size of adenoid) [1]. Nasal endoscopy showed the most common hypertrophy in both the case and control groups was second-degree hypertrophy. Third-degree hypertrophy was more in the case group, with fourth-degree hypertrophy in the control group being zero [5]. 

**Table 3:** X-ray findings of nasopharynx lateral view and nasal endoscopy findings

Findings	Case	Control
X- Ray Nasopharynx		
Grade I	9	10
Grade II	15	25
Grade III	17	13
Grade IV	8	1
Total	49	49
Nasal endoscopy findings		
First degree	8	14
Second degree	18	28
Third degree	17	7
Fourth degree	6	0
Total	49	49

The increase in CHL was directly proportional to the increase in size of the adenoids. The highest degree of hearing loss (32-48 DB) was present with fourth-degree adenoids (Table 4).

**Table 4:** Degree of conductive hearing loss in children in relation to the size of adenoids

Nasal endoscopy finding	Degree of conductive hearing loss in dB
First degree	26-35
Second degree	26-37
Third degree	28-40
Fourth degree	32-48

The odds ratio was calculated to be 5.308 with a confidence interval of 1.98 to 14.10 (Table 5). The risk of having CHL was five times higher in children presenting with third- and fourth-degree adenoids.

**Table 5:** Association between size of adenoids and conductive hearing loss

Size of adenoid	Case	Control	Total
Large adenoid (third and fourth degree)	23 (76.70%)	7 (23.30%)	30
Small adenoid (first and second degree)	26 (38.20%)	42 (61.80%)	68

## Discussion

The total number of participants enrolled for this study was 98, out of which 49 were in the case group and 49 were in the control group. History was taken carefully and all the patients underwent detailed clinical examinations and investigations were done from 2nd January 2017 to 2nd January 2018.

Our study included children aged between 5 and <15 years. The majority were in the age group 5-7 years and the mean age was 7.67 years. In a similar study, 84 patients of CHL with adenoids were found in the 3-10 years age group. The study reported enlarged adenoids in 76.31% of patients between the ages of 3 and 10 [4]. Adenoids appear to be the largest in the age group of seven-year-olds [6].

The male-to-female ratio of patients of adenoids in this series was 1.80: 1, which was in accordance with another study (1.30:1) by Sarwar et al. [7].

The most common symptoms of the patients with adenoid hypertrophy were hearing impairment (79.6%), mouth breathing (63.3%), and nasal obstruction and discharge (69.4%). The findings of this series were consistent with the findings in a study done by Pagella and Sarwar et al. [7,8]. The difference in symptoms like hearing impairment, mouth breathing, nasal obstruction, snoring, headache, and earache between the case and control groups was statistically significant.

In this study, the CHL ranged from mild to moderate degrees according to the World Health Organization i.e. 26 dB to 55 dB [9]. Around 97.10% of case ears had hearing loss between 26 to 40 dB and 2.89% of case ears had a hearing loss in the 41-55 dB range. The mean hearing loss was 31.69 dB. The threshold of hearing found in this series was similar to the finding of a study done by Sarwar et al. [7]. 

Tympanometry is an easy, reliable, and accurate test to detect fluid in the middle ear [4]. In our study, the type B curve was seen in 40.81% and the type C curve in 26.53% of ears. The combined sensitivity of otoscopy and tympanometry of the type B curve is 98%. In 1986, it was observed that adenoidectomy alone produced no peak/peak conversion in 29.8% of children [10].

In this study, adenoid hypertrophy was found to be a possible associating factor in the incidence of CHL and OME. It was also found that there was an increase in the incidence of CHL with the increase in the sizes of adenoids, as 58.33% of patients with enlarged adenoids suffered from OME. 

In the X-ray nasopharynx lateral view, the majority of the case group had grade III hypertrophy while the majority of the control group had grade II hypertrophy. Grade IV hypertrophy was seen in eight (16.32%) cases and one (2.04%) control group. 

Nasal endoscopy showed that the majority of the case group had second-degree hypertrophy (36.73%), followed by third degree (34.69%), first degree (6.32%), and fourth degree (12.24%) and the majority of the control group had second-degree adenoid hypertrophy (57.14%) followed by first degree (28.57%) and then third degree (12.28%). A higher grade of adenoid hypertrophy was more common in the case group and a comparatively lower grade of adenoid hypertrophy in the control group. Fourth-degree adenoid hypertrophy was more common in the case group than the control group which is similar to the study done by Acharya et al. [11]. While relating to the data obtained from X-ray and nasal endoscopy, the findings are quite similar but to accurately diagnose adenoid hypertrophy, nasal endoscopy is found to be far superior to X-ray [12]. 

In our study, it was found that the degree of CHL increased with the degree of obstruction caused by adenoids. The risk of having CHL was five times higher in children presenting with third- and fourth-degree adenoids. Thus, third- and fourth-degree adenoid-causing symptoms can be considered as one of the selection criteria for surgical treatment [11].

The main limitation of this study is that patients did not follow up regularly after surgery. The improvement in the hearing could not be evaluated after medical or surgical treatment of adenoids. So, the benefits of adenoidectomy for the affected children as done in other studies could not be confirmed from this study [13]. 

## Conclusions

In children, adenoid hypertrophy was closely related to CHL. In our study, the mean age of occurrence of adenoid hypertrophy with CHL was 7.67 years. Male children were affected more compared to females. It was found that the increase in size of adenoids was directly proportional to the increase in the degree of CHL. The risk of having CHL was five times higher in children presenting with third- and fourth-degree adenoids. The highest degree of CHL was present with fourth-degree adenoids. Between X-ray nasopharynx and nasal endoscopy, we concluded nasal endoscopy was a superior method for evaluating of patients with adenoids.

In our study, we found that adenoid hypertrophy has a positive correlation with conductive hearing loss in pediatric patients, so proper screening and early management should be done to prevent hearing loss in children. Alongside medical treatment, adenoidectomy is being increasingly used for the treatment of symptomatic adenoids because it has been an effective method of treatment.
